# Investigating the role of socio-demographic variables to psychosomatic symptoms of a greek sample between the two domestic COVID-19 lockdowns

**DOI:** 10.1192/j.eurpsy.2021.1756

**Published:** 2021-08-13

**Authors:** G. Pilafas, G. Lyrakos, V. Spinaris

**Affiliations:** 1 Psychology Department, Cardiff Metropolitan University at City Unity College, Athens, Greece; 2 Department Of Psychiatry, General Hospital of Nikaia “Ag. Panteleimon”, Nikaia, Greece

**Keywords:** Psychological burden, demografic characteristics, COVID-19

## Abstract

**Introduction:**

The COVID-19 outbreak resulted in two respective social and economic lockdowns in Greece. According to international findings pressure and instability may lead to the sense of losing control over the situation, and in retrospect to the escalation of psychosomatic symptoms for the general population.

**Objectives:**

The present study examines whether five socio-demographic variables are significant to the variance of psychosomatic symptoms of the Greek population between the two domestic lockdowns.

**Methods:**

192 participants, of whom 141 were females(73.4%) and 51 males(26.6%), provided their answers between October 5 and November 18, 2020 to the research team of the Psychiatric Unit of the General Public Hospital of Nikaia, ‘Ayios Panteleimon’, in Athens, Greece. The participants were asked about their (i)‘income’, (ii)‘occupation’, (iii)‘residence’, (iv)‘marital status’ and (v)‘education’. Psychosomatic symptoms were measured through the self-reported PSSQ-29 tool (Cronbach’s alpha= .955).

**Results:**

Out of the five One-way Between-participants ANOVAs, none of the five socio-demographic variables showed any significant statistical difference in the level of psychosomatic symptoms.

**Conclusions:**

The study provides some evidence against the protective and harmful role of the socio-demographic variables in psychosomatic health. It is noteworthy, that the conditions were not similar with previous studies. It might be possible that the COVID-19 worked as a phenomenon of mass panic for the Greek sample, and thus no socio-demographic background was either protective or harmful. In conclusion, the present study clearly highlights that none of them had any significant effect to the variance of psychosomatic symptoms for the general population.

**Disclosure:**

No significant relationships.

